# Editorial: World Alzheimer's month: cognitive frailty, Alzheimer's disease and dementia: how do they relate? The role of cognitive reserve

**DOI:** 10.3389/fpsyg.2023.1267306

**Published:** 2023-10-06

**Authors:** Laura Lorenzo-López, Manuel Gandoy-Crego, Leida C. Tolentino

**Affiliations:** ^1^Gerontology and Geriatrics Research Group, Instituto de Investigación Biomédica de A Coruña (INIBIC), Complexo Hospitalario Universitario de A Coruña (CHUAC), Servizo Galego de Saúde (SERGAS), Universidade da Coruña, A Coruña, Spain; ^2^Department of Psychiatry, Radiology, Public Health, Nursing and Medicine, University of Santiago de Compostela, Santiago de Compostela, Spain; ^3^Department of Psychology, Santa Barbara City College, Santa Barbara, CA, United States

**Keywords:** cognitive frailty (CF), dementia, Alzheimer's disease, cognitive reserve, frailty

With the global aging population, understanding the risk factors associated with cognitive decline and dementia has become increasingly important. Physical frailty and cognitive decline are common manifestations of the aging process and are closely related (Borges et al., 2019; Sargent et al., 2020). Both are complex and multifactorial conditions that affect older adults' quality of life and place a burden on healthcare systems. However, the underlying mechanisms linking aging, physical frailty, and cognitive impairment remain unclear.

Cognitive frailty is a clinical syndrome characterized by cognitive impairment due to physical frailty in the absence of concurrent neurological diseases (Kelaiditi et al., 2013; Facal et al., 2019; Ma and Chan, 2020), often leading to an increased vulnerability to dementia and other adverse outcomes. It has been described as a state of reduced cognitive reserve (CR) that is different from physiological brain aging and potentially reversible (Kelaiditi et al., 2013). CR refers to an individual's ability to withstand and compensate for age-related brain changes through adaptive strategies and experiences (Stern, 2002, 2012). Epidemiological studies have suggested that lifetime exposures, including educational and occupational attainment, as well as engagement in leisure activities in late life, can increase CR, potentially delaying the onset of symptoms in neurodegenerative disorders such as Alzheimer's disease (AD) and protecting against frailty (Sardella et al., 2020). However, there is a need to understand the mechanisms that determine cognitive frailty and its relationship to dementia from different points of view. The present Research Topic aimed to shed light on the impact and risk factors of cognitive frailty and dementia and the importance of early detection through close monitoring and targeted interventions.

The results of the study by Gaspar et al. shed light on both protective factors and vulnerabilities associated with cognitive frailty and mental health. The study centered on investigating the influence of CR on mental health outcomes within different cognitive frailty phenotypes in the Portuguese population. These phenotypes aim to capture the heterogeneity of cognitive frailty, considering the varying combinations of cognitive impairment and physical frailty. Higher levels of education and engagement in mentally stimulating activities were identified as protective factors, as they were linked to higher CR and better mental health outcomes. On the other hand, factors such as physical frailty and depressive symptoms were associated with lower CR and poorer mental health. The study revealed that participants with higher CR demonstrated better mental health outcomes across all cognitive frailty phenotypes, highlighting the potential protective role of CR in maintaining psychological wellbeing. Future research should expand upon these findings, examining CR, mental health, and cognitive frailty in diverse populations to identify commonalities and possible cultural variations.

An updated systematic review and meta-analysis focusing on multi-concept frailty (Guo et al.) has shed new light on frailty significance as a predictor of cognitive disorders in late life. Multi-concept frailty is a comprehensive approach recognizing frailty as a complex, multifaceted condition influenced by multiple interconnected domains, including physical, psychological, social, and cognitive factors. This review, encompassing a comprehensive range of longitudinal studies, revealed a robust association between multi-concept frailty and the occurrence of cognitive impairment or dementia. These findings have important implications for clinical practice since identifying individuals at risk for cognitive decline or dementia and addressing the underlying factors contributing to frailty across multiple dimensions can enable healthcare professionals to intervene early and implement targeted preventive strategies.

The study by Alenius et al. suggests that a combination of national and local health records has the potential to provide valuable data for large-scale cognitive screening. These sources can offer information on individuals' health status, medical history, and demographic data, which can be crucial in identifying risk factors and patterns related to cognitive impairment, including AD. Indeed, national registers are unique resources since they provide nationwide coverage of real-world data on dementia diagnosis. Currently, the process of collecting and analyzing data from these sources may be time-consuming and laborious, often requiring manual extraction and compilation of relevant information. However, advancements in technology, particularly in the field of data analytics and electronic health records, hold promise for improving the efficiency of the process in future, improving early detection, risk assessment, and the development of preventive strategies for cognitive impairment and dementia.

Finally, in a minireview, Ávila-Villanueva et al. described the main structural and functional brain changes found at different stages of AD, focusing on alterations that occur during the prodromal stage. AD is the major cause of dementia, and its neuropathological process occurs over a period of years. Early detection methods, such as neuroimaging techniques (e.g., magnetic resonance imaging, MRI; positron emission tomography scans, PET) and biomarker analysis (e.g., cerebrospinal fluid analysis), can aid in the identification of these brain alterations. Detecting brain changes before clinical symptoms emerge, when individuals may still be cognitively normal or experiencing mild cognitive impairment, can be crucial for early intervention and the development of preventive strategies aimed at slowing down dementia progression. Further research should indeed focus on identifying factors that may contribute to the reversion or preservation of cognitive function in individuals with AD. Factors such as genetic variations, lifestyle factors (e.g., regular physical exercise, leisure time activities, nutritious diet, and cognitive stimulation), social engagement, and other individual characteristics may play a role in promoting cognitive resilience and should be investigated further.

The scientific contributions of the Research Topic suggest that a holistic approach is essential to capture the clinical and biopsychosocial complexities of frailty and dementia. Healthcare professionals need to consider not only the physical aspects of frailty but also the psychological, social, and cognitive dimensions. Comprehensive assessment tools can aid in evaluating these various dimensions of frailty, helping clinicians develop individualized care plans that address specific vulnerabilities and promote overall wellbeing. Although promoting CR emerges as a promising approach to prevent or delay cognitive frailty and dementia, the exact neural mechanisms underlying their relationship are not fully understood and should be further explored.

## Author contributions

LL-L: Conceptualization, Writing—original draft, Writing—review and editing. MG-C: Conceptualization, Writing—review and editing. LT: Conceptualization, Writing—review and editing.
